# Temporal and spatial variations in local sex ratios in a suburban population of the European green toad *Bufotes viridis*

**DOI:** 10.1186/s12862-023-02106-0

**Published:** 2023-03-17

**Authors:** Martina Staufer, Stephan Burgstaller, András Horvath, Lukas Landler

**Affiliations:** 1 Lindenbauergasse 13, 1110 Vienna, Austria; 2 Institute of Zoology, Department of Integrative Biology and Biodiversity Research, University of Natural Resources and Life Sciences Vienna, Gregor-Mendel-Strasse 33, 1180 Vienna, Austria

**Keywords:** Anura, Amphibian migration, Pond-breeding, Population demographics, Prolonged breeder, City, Austria

## Abstract

**Background:**

Sex ratios of animal populations are important factors of population demographics. In pond-breeding amphibians, the operational sex ratio (OSR) among the breeding population is usually male-biased. Also, in European green toads (*Bufotes viridis*), males usually outnumber females at breeding sites, while the sex ratio of the total adult population (ASR) is assumed to be balanced. It has been suggested that sex-specific breeding behavior causes male-predominance at the breeding sites. We used a dataset of 5 years of street patrols to test this hypothesis. For this we analyzed local sex ratios of green toads in terrestrial habitats and at two artificial breeding ponds. We expected temporal and/or spatial changes of local sex ratios which would indicate sex dependent differences in breeding behavior.

**Results:**

Overall observed ASR among 2111 green toads, counted in the course of street patrols from 2016 to 2020, was slightly male-biased (ASR = 0.56, annual ASRs = 0.49–0.63). Based on the data of 1631 toads (920 males, 711 females) captured within a radius of 300 m around nine main breeding sites, temporal and spatial variations in local ASRs were evaluated. Resulting values were compared to the calculated OSR at two artificial breeding ponds in 2021 (645 adult: 553 males, 92 females). Estimates predict more equally distributed females and males prior to the main breeding season. During breeding season, males predominated at both breeding sites (B1: 0.83, B2: 0.89), whereas females are estimated to outnumber males in terrestrial habitats. Proportions of females highly significantly increased with advancing time of the year and increasing distance to the breeding sites. While males tended to accumulate in proximity to water bodies, females dispersed soon after breeding to more distant areas.

**Conclusions:**

Observed sex ratios in the studied green toad population changed with time and sampling site, deviating from the population-wide sex ratio. Expanding sampling effort in amphibian conservation assessments in time and space, i.e., outside the main breeding season and away from the breeding sites, would be important to encompass such variations.

**Supplementary Information:**

The online version contains supplementary material available at 10.1186/s12862-023-02106-0.

## Background

Assessing population demographics is key to understanding animal ecology, and is also important for species conservation [1]. To analyze demography, among other factors, sex ratio is fundamental, and needed to estimate population size and dynamics [2, 3]. Fisherian theory predicts sex ratios of 1:1 if the production of males and females is of equal cost [4, 5]*.* In many examples, sex ratios at birth are indeed balanced, however, can change at age of maturity [e.g., 6]. In Amphibian populations, sex structure can be altered in all life stages by abiotic and biotic factors. For example, exposure of larvae to different kinds of chemical pollution like heavy metals can result in a higher proportion of females compared to unaffected populations [e.g., 7]. Amphibians are also sensitive to endocrine disrupting compounds leading to phenotypic sex reversal of genetic males during development [e.g., 8]. Temperature regime (reviewed in [9, 10]) or a sex-specific mortality in road traffic [11, 12] might also bias the sex ratio.

The majority of sex ratios of pond-breeding amphibians recorded at breeding sites are male-biased (e.g. [13–16], but see [17] for an exception). The question of whether the proportions of males and females at breeding sites (operational sex ratio—OSR) correspond to the population wide situation of adults (adult sex ratio—ASR) has been discussed controversially [e.g., 18–20]. Sex differences in age at maturity [21–23] and longevity [13, 14] could change the OSR and the ASR. In contrast, sex dependent breeding behavior could change the OSR but would not influence the ASR. Such behavioral differences have been reported in many amphibians and include different arrival and residence time at the breeding sites [24], differing breeding frequencies (skipping of breeding seasons) [16, 25, 26] and site fidelity [e.g., 27, 28]. Studies of pond-breeding amphibians are usually focused on the breeding sites, which means that mostly OSRs are reported. One reason for this is that many species are difficult to detect in their terrestrial habitats. However, to estimate the ASR, in such a situation, one would need capture-recapture data throughout all seasons and all habitats [e.g., 29].

Several studies provided statistical analyses to explain male predominance at breeding sites, but there is still a lack of profound estimates of female abundances and hence, ASR in most species and populations. We are aware of only 2 studies which have documented the ASR of pond-breeding anurans apart from the breeding season. As shown by Green [29], observed annual OSRs in the explosive-breeding Fowler’s toad (*Anaxyrus fowleri*) varied substantially between years, and estimates by capture-mark-recapture (CMR)-methods predicted a mean 5.34 to 1 male-dominance at the breeding sites. In contrast, observed and estimated sex ratios across breeding and terrestrial habitats were substantially less biased and balanced, respectively (1.43 vs. 1.03 males per female). Observations among hibernating Tibetan frogs (*Nanorana parkeri*) revealed similar results (1.26:1), though no CMR-methods were used [30].

We analyzed a 5-year (2016–2020) time series of street patrol data of European green toads (*Bufotes viridis*) to gain some further insights into this understudied issue. For our analysis, we assumed that local sex ratios of road-crossing toads are representative demographic samples of the population—including reproductively active and inactive adults. These results were compared to the observed OSRs of two central water bodies’ breeding communities. The green toad is a prolonged pond-breeding species [sensu 18]. Anurans with an extended breeding season are physiologically able to reproduce continuously as long as environmental conditions are favorable [31]. Reproduction periods of entire breeding communities usually span over several consecutive months [32]. Since green toads live a terrestrial life apart from reproduction, during breeding migration entire reproductive populations circulate between terrestrial habitats and aquatic breeding sites.

Previous studies have shown a wide range of observed sex ratios for green toad populations, with numbers of males exceeding females by up to 8.5 times at breeding sites [32–34]. Observed proportions of males tended to be higher when determined by capture at breeding sites (0.52–0.88) [35, 36] than by capture at drift fences around them (0.5–0.79) [36, 37]. As part of two separate studies toads were sampled also away from breeding sites: Sinsch et al. [35] found both 21 males and females in daytime hides in up to 1 km distance to a pond. Beckmann et al. [38] determined a male proportion of 0.6 at fenced breeding sites combined with a 1.2 km long linear fence. Genetic and histological sexing of metamorphs of green toads raised under laboratory conditions revealed 12 males and 13 females out of 25 individuals [8].

A common assumption is, that ASR is 1:1 in *B. viridis* populations but sex-specific differences in breeding behavior lead to male-predominance at the breeding sites [32]. This assumption would be supported by our study if (1) the sex ratio was female-biased further away from the breeding sites and (2) observed female proportions changed during the breeding season. In contrast, if sex ratios at breeding sites represent overall ASR, no significant temporal or spatial trends are expected.

## Methods

### Study species and study site

The European green toad, a typical pioneer species, prefers open landscapes and is behaviorally well-adapted to semi-arid to arid conditions. In Central Europe, green toads often inhabit cultivated land in warm climate (reviewed in [32]). Populations in human settlements are common and the species may even occur in large cities [34, e.g., 39]. However, today many populations are declining or disappearing due to further intensification of agriculture and urbanization [32]. One major threat is an ongoing habitat loss, especially of breeding sites [32]. This is despite the fact, that green toads can reproduce in a variety of different water bodies, ranging from shallow temporary puddles to large permanent ponds [32, 40].

**Fig. 1:**
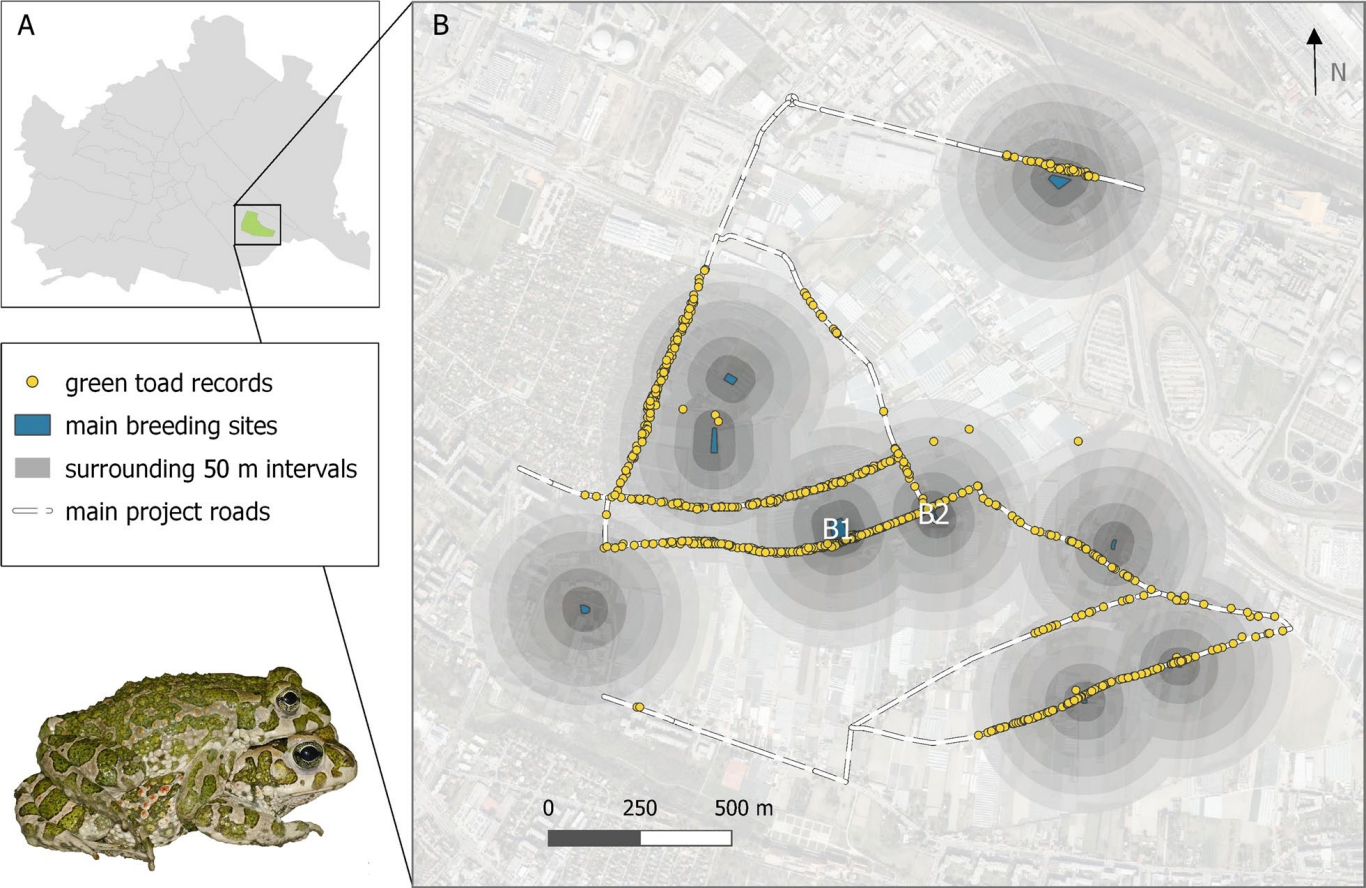
Study site and spatial distribution of adult green toads *Bufotes viridis* during breeding seasons 2016–2020. (**A**) Map of Vienna, Austria (**B**) Map of the “Simmeringer Haide” including individual observations (n = 1631) within 300 m of 9 main breeding sites (B1 and B2 sampled in 2021). Gray circles around each of the water bodies indicate 300 m radiuses split up in 50 m intervals. Maps prepared in QGIS [42]

The surveyed green toad population is located in the “Simmeringer Haide” in the south-eastern outskirts of Vienna, Austria (16.442° E, 48.167° N; Fig. 1A, B). This traditional farmland provides a core area of about 310 ha of suitable habitat for the species. Since the late twentieth century, the area is dominated by greenhouses and polytunnels, whereas open arable land is limited to small patches [41]. Several roads run through the area, making road mortality a significant hazard to amphibians. In this former part of the Danube floodplain, no persistent natural water bodies have been preserved. Large artificial rainwater collection basins are the most important breeding sites today [39]. One of them is B1 with a surface area of about 1800 m^2^ and a constant water depth of at least 3 m. In contrast, B2 is a shallow concrete basin with a mean surface area of 160 m^2^ and a water depth of max. 0.15 m, that desiccates periodically (during breeding season 2021 from end of April to mid-May).

### Data collection

Toads were captured during a roadkill mitigation project from 2016 to 2020 within a street network of approximately 10.8 km. On 30 to 38 nights per breeding season, roads were slowly patrolled either by foot, bicycle, or car by at least two persons, mainly MS assisted by volunteers. Green toads approaching the, or in close proximity to, roads were removed from danger zones and individually registered by photographing their dorsal patterns. Documentation comprised date, time, locality (coordinates), status (alive or dead) and, if possible, age class (juvenile, immature, adult) and sex. Basic data were occasionally supplemented by comments, e.g., amplexus, conspicuousness of nuptial pads, release call, increased abdominal size (egg-carrying females) or spawn in overrun females. Toads were photographed in standardized small buckets, so that in case of doubt the size could be estimated retrospectively as one key factor for the assignment to an age class. The denoted sex determination was checked again when images were combined with the data. Deviations due to different skills of volunteers can therefore be ruled out. Finally, 82% of all alive and 50% of the dead toads could be sexed. Data from adult individuals with determined sex and precise location, captured within a 300 m radius of nine main breeding waters, were used in the analysis (Fig. 1B). Collecting surveys were not carried out systematically, however we surmise that they represent pseudorandom samples of the local toad assemblages, including resident as well as migrating individuals.

Furthermore, two centrally located breeding sites were sampled during breeding season 2021 (25.3.–16.7.2021) on 12 (B1) and 18 (B2) nights, respectively (Fig. 1B). Recorded toads were handled such as described above, i.e., adult individuals were sexed, and pictures were taken. The total individual numbers of males and females per site were used to calculate observed OSRs. For both roads and ponds, double counting of individuals per study time interval (month or year) was avoided by comparing pictures of the dorsal patterns with the software HotSpotter [43], which performed best with green toads in a pilot study [44], and the follow-up version IBEIS [45].

### Sex and age determination

Adult green toads exhibit a minor sexual dimorphism with morphological differences in body size and coloration, whereby females tend to be larger [36] and more contrastingly colored [46]. Some secondary sex-specific traits allow an accurate and easy identification in most cases. During breeding season phenotypic males or females can be best determined by the presence / absence of nuptial pads on the thumbs and muscular forearms [32]. In some individuals with doubtful external characteristics, the determination had to be confirmed by the specific male and female release call [47, 48]. In general, it can be assumed that those animals that could be clearly sexed were also adult or vice versa, individuals with no clear identifying features and/or small size were classified as indeterminate and immature, respectively. Using this approach a few small females may have been classified as immature erroneously as both sexes may mature in their 2nd year of life at a minimum size of around 50 mm snout-vent length [e.g., 36].

### Statistical analysis

All statistical analyses were calculated in R [49]. Overall recapture rate was very low during the entire study period: only 3.1% of all individually recognized toads were recaptured at least once. The low annual recapture rates did not allow to apply capture-recapture models to estimate sex ratios. Alternatively, we used the proportion of females for each of the monthly sampling events (at each sampling location) as the response variable to analyze the relative abundance of females. For the full generalized linear mixed effects model (GLMM) the independent fixed effect variables were year, month, street, and distance to the next breeding pond (in 50 m increments) and all their two-way interactions. In addition, year, month, and street were added as random effects. After initial error distribution comparisons we decided to use a Tweedie distribution (as parameterized in the glmmTMB package [50], i.e. V = φμp; with variance V, dispersion parameter φ, predicted mean μ, and the power parameter p restricted to 1 < p < 2) with zero inflation (zero inflation model: 1|month), fitted with the function glmmTMB [50]. We then performed an AIC based model selection with the function buildglmmTMB of the package buildmer [51]. The model predictions were calculated with ggpredict and plotted with the plotting functions of ggeffects [52], ggbarplot [53] and patchwork [54]. Finally, the best statistical model included the fixed factors month, distance, and their interaction; year and street were included as random effects. An ANOVA table was generated from the best model using the function Anova.glmmTMB [50].

## Results

During the 5-year study period a total of 2111 adult individuals of specified sex were recorded on the roads from mid-March to the end of July. Total captures per breeding season ranged from 224 (2017) to 896 (2019) alive and dead toads, 25% of all counts were roadkill. ASRs, expressed as the proportion of males in the adult entity, among toads captured at roads in any one year ranged from 0.49 to 0.63. Throughout the study period 56% of all records were males.

Annual spring migration started around mid-March. First road-crossing toads, in between winter hibernation and the regular breeding season, were mostly male. Generally, main breeding season lasted from April to June. However, outstanding early arrival of females at the breeding sites and first spawning events have been observed in two years (17.3.2019 and 12.3.2020) when above-average temperatures in late winter led to a particularly early start to the season [55].

**Fig. 2:**
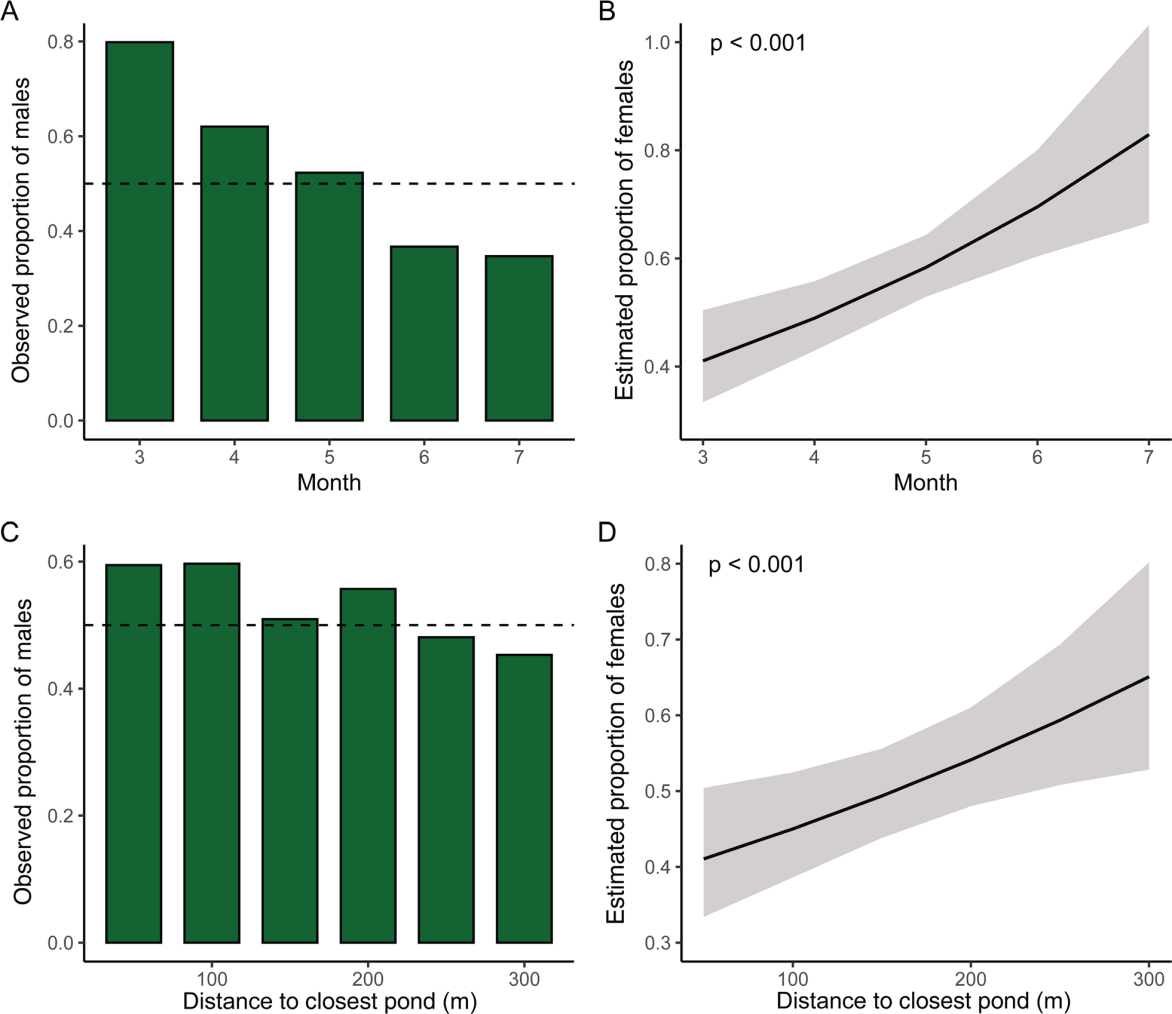
Observed proportions of green toad *Bufotes viridis* males during breeding seasons 2016–2020 per month (**A**) and per 50 m distance interval to the closest breeding site (**C**) compared to predicted trends in the proportion of females over time (**B**) and distance (**D**)

A total of 1631 adult toads (920 males, 711 females) were captured within 300 m of the nearest breeding site. Calculated ASR was 0.56 males per adult (1.3 males per female). Total captures increased from March to May and decreased afterwards, reflecting main migratory activity. Observed proportions of females at the capture locations were lowest in March and close to the breeding sites (Fig. 2A, C). Overall observed ASRs were most balanced in May (mid breeding season) and within the 150 m interval (101–150 m). From May onwards females outnumbered males in all distances but close to the breeding sites (up to 50 m). Relative frequency of females highly significantly increased with ongoing season and with increasing distance to the breeding sites, with a trending interaction between distance and month (Table 1). The model predicts higher proportions of females than males at each of the sampling sites from April onwards (note: this does not equal absolute numbers of females), indicating more evenly distributed females and locally clustered males (Fig. 2B, D).

**Table 1:** ANOVA table of the model results with female proportion as the response variable

Factor	χ^2^	p
Month	20.32	< 0.001
Distance	16.12	< 0.001
Month by distance	3.15	0.076

The ‘proportion females’ used as the response variable in this analysis represents the proportion of females that can be found at one given month at one of the sampling locations (i.e., the absolute numbers of females and males can vary substantially)

Males were substantially more predominant at the two breeding sites (B1: 286 males, 60 females; B2: 267 males, 32 females) in comparison to the data from the street patrols. Based on the number of captured individuals, i.e., ignoring potential sex-specific capture probabilities, OSRs were 0.83 (4.8 males per female) at the permanent basin B1 and 0.89 (8.3 males per female) at the temporary water body B2.

## Discussion

Our study illustrates that survey data on sex ratios are highly context-dependent in pond-breeding anurans. Spatial aggregations and distributions of individuals vary between the breeding and the non-breeding seasons. In prolonged-breeding species, reproduction is associated with long-lasting migrations, so that during the breeding season the membership of individuals in aquatic or terrestrial groups is asynchronous, staggered, and unpredictable in time. Discrete counts are temporary snapshots, where sex ratios can be highly variable, particularly along migratory routes and where abundance is generally low.

The differences in the observed sex ratios at both breeding sites (OSRs = 0.83 and 0.89) and in the surroundings (ASR = 0.56), are consistent with the assumption that high male predominance at breeding sites does not reflect the effective population composition. It has been shown in CMR studies that estimated amphibian sex ratios tend to be less skewed than counts suggest, and relative abundance of males can easily be overestimated [e.g., 56], even outside the breeding season [29, 57] and regardless of the species’ biology. Based on our census data and analysis there is strong evidence, that the ASR in our studied *B. viridis* population is close to 1:1. The following results underline the likelihood of parity in the total adult entity: (a) annual sex ratios among all captured toads during the 5-year study period varied around 0.5; (b) model estimates predict female predominance in the surroundings, while males tended to cluster at and near the ponds, which likely levels out overall; (c) estimated adult female proportions early in the season suggest that males and females are equally distributed over the surveyed area before the main breeding migration causes severe relocations among the entire reproductive population.

Significant changes of local sex ratios indicate that males and females behaved differently in terms of the timing of arrival at, and the departure from, the breeding sites. Asynchrony in migration initiation, with males arriving first at the breeding sites, is a common and well-known phenomenon in pond-breeding anurans [e.g., 58] and also in *B. viridis* [32]. Among other factors, sex has been found to already affect the timing of hibernation in amphibians, whereby males emerged from hibernation earlier than females [59]. In the case of prolonged-breeding species in particular, differences in residence duration are considered the main influence on the sex ratio within the breeding communities [e.g., 36]. Individual *B. viridis* males may stay for several consecutive weeks or visit the breeding sites repeatedly, whereas females usually arrive only once, stay just as long as necessary to complete mating and oviposition, and then depart [32].

Estimated steep increases of female proportions with time and distance indicate that after breeding, females have a head start in dispersing and also migrate to more distant areas. Differences by migration distance have been found in several explosive-breeding amphibians ([e.g., 60–62] but see [21, 63]), and also in the prolonged-breeding natterjack toad *Epidalea calamita* [27] but not in two *Pelophylax* spp. [64]. One hypothesis is that males overwinter close to the breeding sites to be able to return there earlier in the following season, which is thought to enhance their reproductive success [65, 66]*.* But this is probably less relevant in species with asynchronously and prolonged breeding females. Furthermore, diverging post-breeding migration distances have been associated with differences in site fidelity [27, 67] and habitat selection [68].

Low individual numbers within the 300 m distance interval suggest that mainly resident toads were captured and the reoccurring emigrations of reproductive adults, among which the sex ratio was male-biased, rather than post-breeding remigrations, accounted for the increasing female bias in sex ratios. Farther away from breeding sites, more females may have not participated in breeding activity. Studies across a range of amphibian species have shown facultative breeding [25, e.g., 68]. Particularly females [16, e.g., 69], but also males [29, e.g., 70] and young adults [71] have been found to skip one or more breeding opportunities. Iteroparity can be limited by several factors, including energy costs of reproduction [72, 73], and unfavorable environmental conditions like the lack of rain and breeding sites [26, 29]. While later maturation of females versus males is common in anurans, in *B. viridis* both sexes can mature in their 2nd year of life [36, 74]. However, many females delay maturity to their 4th year of life [36]. Substantially more non-breeding or sexually immature females than males would also contribute to the predominance of the latter at the breeding sites. It is therefore very likely, that more males than females entered the surveyed breeding communities, though the magnitude of the skewness is expected to be severely overestimated by counts.

Sex-biased physiological demands, habitat preferences, behavior, visibility, and vocalization of males can lead to diverging capture probabilities of different sexes in amphibians [29, 57]. We observed male accumulation along one road section that passes right next to a water body. Occasionally, several males remained there presumably to watch for incoming females, increasing their chance of being captured. Besides that, there were no apparent differences in road-use between the sexes. However, sex differences in capture probability would have biased observed abundances but would not have influenced temporal and spatial trends. Model predictions indicate high female proportions, especially where generally low individual numbers were recorded. Hence, while female proportions at locations further away from the breeding sites are high, this does not translate to equally high counts. Despite toads’ high detectability on the roads, capture probability of both sexes is supposed to be low, as the road sections only account for a minor fraction of the survey area. Recaptures were very rare and could not be improved substantially even by increased efforts in 2019. Low recapture rates are usually associated with a large population size and/or a high mortality rate. In addition, many individuals probably only crossed the roads once or twice on their way to and from breeding sites, which would also explain low recapture rates within single years. Previous studies have shown that green toads are very mobile [32], for example, a maximum daily migration distance of 558 m has been observed in a German population [75]. Thus, the nocturnal toads could have covered the studied environment of 300 m around the breeding sites within one night.

Considering the many factors, that can affect sex ratios in amphibian populations, it seems quite unlikely that sex ratios in different populations are exactly the same. The ASR might be closely related to past and present environmental conditions that influenced the development or longevity of individuals. Variations can potentially occur between subpopulations or even breeding assemblages that breed in different bodies of water. In urban populations in particular, deviations may be expected due to various anthropogenic factors, such as pollutant, that may change rapidly [7, 8, 10]. To understand sex ratios in amphibian populations, intensive long-term studies are necessary. These include sampling over the entire breeding season, if possible, longer, and surveying breeding sites as well as their surroundings (> 100 m radius). Individual recognition can help to correct for double counting or can even be used for sex specific population modelling, in case of sufficient recaptures. Otherwise, any sex-ratio estimates will be skewed by the asymmetries in space and time.

## Supplementary Information

**Additional file 1.** R code and data.

## Data Availability

All data are included in the article text as well as its additional information files. Data and R script for analysis available in the Additional file 1.
